# Self-Reported Dental Caries by Mexican Elementary and Middle-School Schoolchildren in the Context of Socioeconomic Indicators: A National Ecological Study

**DOI:** 10.3390/children8040289

**Published:** 2021-04-08

**Authors:** Juan Fernando Casanova-Rosado, Alejandro José Casanova-Rosado, Mirna Minaya-Sánchez, Juan Alejandro Casanova-Sarmiento, José Luis Robles-Minaya, Sonia Márquez-Rodríguez, Mariana Mora-Acosta, Rosalina Islas-Zarazúa, María de Lourdes Márquez-Corona, Leticia Ávila-Burgos, Carlo Eduardo Medina-Solís, Gerardo Maupomé

**Affiliations:** 1 School of Dentistry, Autonomous University of Campeche, Campeche 24039, Mexico; juanf_casanova@yahoo.com.mx (J.F.C.-R.); ajcasano@uacam.mx (A.J.C.-R.); juancasanova1192@gmail.com (J.A.C.-S.); jlroblesm17@gmail.com (J.L.R.-M.); 2 Academic Area of Dentistry, Health Sciences Institute, Autonomous University of Hidalgo State, Pachuca 42160, Mexico; cdsoniamr@hotmail.com (S.M.-R.); mtramarianamoraa@hotmail.com (M.M.-A.); riz751@hotmail.com (R.I.-Z.); lmarquez@uaeh.edu.mx (M.d.L.M.-C.); 3 Health Systems Research Center, the National Institute of Public Health, Cuernavaca 62100, Mexico; leticia.avila@insp.mx; 4 Advanced Studies and Research Center in Dentistry “Keisaburo Miyata”, School of Dentistry, Autonomous University of State of Mexico, Toluca 50000, Mexico; 5 Richard M. Fairbanks School of Public Health, Indiana University/Purdue University, Indianapolis, IN 46202, USA; gmaupome@iu.edu; 6 Indiana University Network Science Institute, Bloomington, IN 47408, USA

**Keywords:** oral health, dental caries, self-report, schoolchildren, Mexico

## Abstract

The objective of the present research was to quantify the association between dental caries self-report and socioeconomic indicators in Mexican children. An ecological study included a self-report of dental caries in schoolchildren enrolled in public elementary and middle schools derived from the National School Health Survey. A total of 73,560 schoolchildren (representing 19,745,366 students) aged 5 to 16 years were included. Socioeconomic variables included were scales depicting physical characteristics of housing, purchasing power, etc. used in national surveys in Mexico to measure deprivation, poverty, and income inequality in official data. Data were analyzed in Stata using Spearman’s correlation test. For the most part, no association (*p* > 0.05) was found between caries self-report, socioeconomic variables, or the Gini index. However, caries self-report in elementary schoolchildren and total (elementary + middle-school) schoolchildren groups was positively correlated (*p* < 0.05) with two poverty variables: extreme poverty by income (value of personal food purchases per month) and poverty by income (value of personal food and non-food purchases per month). National data for dental caries self-report were associated—at the ecological level—with a few socioeconomic indicators but not with most of the usual and customary indicators used in national surveys in Mexico.

## 1. Introduction

Oral diseases affect a large number of people around the world. Findings from the 2017 Global Burden of Disease study show that oral diseases remain a major challenge. Around the world, there were 3.5 billion cases of oral conditions: 2.3 billion had untreated caries in permanent teeth and 532 million had untreated caries in deciduous teeth [1]. While in Latin America there is only a small number of countries that report dental health status with nationally representative samples [2], dental caries is the most prevalent oral disease [3]. In Mexico, the situation is similar, with low utilization of preventive dental services and high prevalence of caries and treatment needs in children and adolescents [4,5,6,7,8,9,10]. In general, about half of individuals between 6 and 12 years of age present caries lesions in any tooth of either primary or permanent dentition [4,10], with consequences in terms of pain, suffering, functional impairment, and reduced quality of life. Access to oral care is limited in many developing countries; teeth are often left untreated or extracted [3,4,6,11,12,13,14,15]. Tooth loss is relatively common in the young population: research reports from Mexico indicate figures between 13.5% and 34.5%, with average missing teeth ranging from 0.31 ± 0.92 to 0.46 ± 1.13 [16,17,18,19,20].

In planning health services, levels of health/disease in the population should be measured to determine their needs. Health needs are defined as the degree of health disease that potential users of health services experience [21]. To understand oral disease patterns, it is crucial to assess and monitor prevention and control using national and global estimates to inform dental health policy planning and evaluation [1]. Despite a decline in dental caries in developed countries over the past decades, dental health inequalities are still present. Socioeconomic inequalities in health may arise from diverse access to key resources—knowledge, money, and power—leveraged to attain health and avoid disease. Many causes of inequalities are difficult to identify, in part because they change in relation to their social context [22]. Several studies found significant associations between lower socioeconomic status and higher risk of dental caries [23,24,25,26,27,28,29]. Social factors influence variables associated with caries through modifying living conditions of the individual; it is feasible to gain a more accurate understanding of caries distribution through examining such a relationship. Socioeconomic inequalities in dental health have been observed between countries, with more developed countries having a lower burden of untreated dental caries [1]. In Mexico, dental health is affected by a complex array of factors, including poor access to preventive and rehabilitative care—on account of costs but also of limited information about the importance of oral health [22,30,31]. Stratification of data dimensions is generally accepted in health research, acknowledging the need to go beyond analyses limited to the purely individual level [32]. The present epidemiological study followed an ecological design, where data are aggregated. In ecological studies, the focus of observation is a population or community: condition rates and exposures are measured, and their relationships are examined. Ecological studies (correlational studies) allow the mapping of diseases and risk factors, large-scale comparisons, and contrasts across public health strategies [33,34]. The aim of the present study was to quantify the association at the ecological level between various socioeconomic indicators and dental caries self-report in Mexican elementary and middle-school schoolchildren.

### Context of Dental Services in Mexico

The structure of the Mexican health system is fragmented and largely driven by employment status [35]. The publicly funded health care providers serve mainly two groups: families of government workers who have insurance funded by the government, and those families having one or more members who work in the private sector. The Army, the Navy, and the national oil company constitute a separate subsystem, which provides services with greater coverage. The Mexican government implemented in 2003 the *Seguro Popular* (SP), which is a publicly funded health care plan that provided partial coverage; it was funded by federal and state governments as well as by household contributions. For households in the lowest three income deciles, SP was free of charge. SP has been recently discontinued. Only salaried earners and their families (≈50 million people, about half of the population) have access to clinical services financed by third-party contributions from employees, employers, and government. The rest of the population has variable access. Such large groups comprise the self-employed, the unemployed, agricultural workers, and generally those with low income [36]. Dental services in the publicly funded health care system are underdeveloped and mostly limited to basic actions such as caries prevention, placement of sealants, fluoride topical applications, as well as simple dental restorations such as glass ionomer, resin/composites or amalgam fillings, or undertaking tooth extractions. Most clinical specialty treatments are not covered [37]. The topical applications of fluoride are occasionally covered by publicly funded health insurance services or in health promotion campaigns carried out in schools. Multiple access and coverage barriers in dental care exist in a health system as complex as Mexico’s [38]. Publicly funded health care providers offer a limited set of services that tend to be restricted to urban populations and are affected by long waiting times [39]. The level of coverage for publicly funded dental services is approximately 50% of the population [40]. The ability of households to pay for dental care services is an important limiting factor, with those lacking access to publicly funded dental services being at a disadvantage [41]. Dental services are delivered to a very large extent on a fee-for-item basis that requires out-of-pocket payments from the patient. A largely unregulated system, under the control of dental professionals, leaves costs subject to shifts in the dental marketplace. Finally, many public and private universities across the country have dental clinics that offer services to the general population at prices far below those of private practice [6,38,41,42]. 

According to federal guidelines (Norma Oficial Mexicana), the use of topical and community-based fluorides is regulated across the country. Population level coverage is attained through using fluoridated domestic salt (250 ppm F), except in some areas where endemic fluorosis has been identified. Only in those areas is it feasible to administer topical fluorides, either in public health campaigns or professional care: after three years of age, fluoride topical agents such as gel can be used under professional supervision. A similar level of supervision is ascribed to fluoride prophy toothpastes; fluoride varnish is only employed in high-risk cases, together with a comprehensive treatment plan. Fluoride mouth washes are contraindicated in children under 6 years of age, and they ought to ideally use fluoride toothpaste at 550 ppm fluoride. Toothpastes at 0.551% to 1.5% (551 a 1500 ppm) fluoride concentration should be allowed only after 6 years of age. If their use is inevitable in young children, then a toothpaste serving should be smaller than a pea (5 mm^3^), and expectoration must be encouraged [43]. Together with the sparse pre-school system, elementary and middle schools are included in quarterly functions that emphasize oral health: dental plaque disclosure, tooth brushing technique, oral hygiene, and dental education presentations for younger children. For older children, demonstrations of dental floss use and fluoride rinses are added to the basic program [44]. There is unfortunately no objective evidence of the impact, consistency, or effectiveness of such interventions. 

## 2. Materials and Methods

### 2.1. Study Design

This is an ecological study based on a dental caries self-report conducted in public schools in the 32 states of Mexico. A multi-group ecological study was conducted [45]: this is a generally accepted epidemiological analytical approach that quantifies the association between average exposure levels and disease frequency among various groups (commonly geopolitical groups). 

Data pertained to children participating in the National School Health Survey 2008 (ENSE). Schoolchildren attending public elementary and middle schools in the country were included in the study. The age range for elementary school children was 5 to 12 years of age, and for middle school children, it was 12 and 16 years of age, with some variations due to early entry to school or school year delays [46].

### 2.2. Description of the Origin Survey

Parents or guardians were asked to sign a letter of consent; they were interviewed to collect information related to the child’s health. The main survey was directly administered to each student; those aged 10 years and older answered the survey on their own.

### 2.3. Study Variables

For the present analysis, caries self-report by parent–guardian or child, as appropriate, was obtained. Socioeconomic variables on deprivation and poverty were derived from the standard battery of measures used at the federal and state level by the National Population Council (CONAPO) [47]: (1) percentage of population aged 15 years or more who were illiterate, (2) percentage of population aged 15 years or more who had not completed elementary school, (3) percentage of occupants in private dwellings without piped water supplies, (4) percentage of occupants in private dwellings without sewage or standard urban sanitation, (5) percentage of occupants in private dwellings without electricity, (6) percentage of occupants in private dwellings with dirt floors (i.e., not covered by durable materials such as tile or concrete), (7) percentage of occupants in private dwellings deemed to be living in overcrowded conditions, (8) percentage of population in localities with fewer than 5000 residents, (9) percentage of population with a maximum income level below two minimum wages (in 2008, one minimum wage was $1577.7 Mexican pesos per month, or US$141.57 dollars per month), (10) and the Deprivation Index, which is calculated from the previous indicators. 

In addition, three more variables were used in the study: two poverty indicators, which were the extreme poverty line by income, which is equivalent to the value of the food basket per person per month, and the poverty line by income, which is equivalent to the total value of the food basket and the non-food basket per person per month. In addition, we included the Gini index. This is a measure of income concentration: it is a number between 0 and 1, where 0 corresponds to perfect equality (everyone has the same income) and where the value 1 corresponds to perfect inequality (one person has all the income and the rest have none). These ecological data were obtained from another federal agency: the National Council for the Evaluation of Social Development Policy (CONEVAL) [48].

### 2.4. Statistical Analysis

Data from dental caries self-report and socioeconomic indicators were estimated for each of the Mexican states. Our analytic strategies were guided by results from bivariate analyses. A multivariate analysis could not be undertaken because only a minority of deprivation and poverty indicators and the Gini index were significant in the (correlation) bivariate analyses. The only two indicators that reached statistical significance were those pertinent to poverty levels. In the present report, only correlations were calculated using Spearman’s correlation tests in Stata 14.

### 2.5. Ethical Considerations

This study was based on secondary data obtained from a publicly available dataset. In the original national survey, informed consent was obtained from the parents/guardians of the participants.

## 3. Results

The ENSE is a nationally representative survey that is carried out in Mexico’s 32 states. A total of 644 public schools were selected; information was collected from 73,560 schoolchildren, representing 19,745,366 students enrolled in elementary and middle school levels. A total of 43,316 elementary and 30,244 middle school students filled out the surveys. Ages spanned from 5 to 16 years of age.

Nationally, 48.7% of elementary school students reported having teeth with caries. Chiapas (25.9%), Sonora (32.4%), and Querétaro (36.5%) had the lowest self-reported caries prevalence, while Tlaxcala (58.4%), Estado de México (59.7%), and Michoacán (63.0%) had the highest (Table 1). Among middle school children, 35.1% reported having teeth with caries, those in Guanajuato (24.9%), Tamaulipas (25.4%), and Campeche (27.2%) reported the lowest self-reported caries prevalence, while the highest were in Aguascalientes (44.1%), Hidalgo (49.5%), and Tlaxcala (51.4%) (Table 2). Table 3 presents the prevalence by state jointly for elementary and middle students: Chiapas (29.4%), Sonora (33.5%), and Guanajuato (33.8%) had the lowest caries prevalence estimates, while the State of Mexico (56.3%), Michoacán (56.4%) and Tlaxcala (57.1%) had the highest.

Spearman’s correlation analyses showed no association (*p* > 0.05) between caries self-report and the socioeconomic variables that make up the Deprivation Index and the Gini index. When caries self-report was contrasted with poverty and extreme poverty, it was found that caries in elementary school children was positively correlated (*p* < 0.05) with both poverty variables (Table 1): when poverty and extreme poverty increased, caries self-report also increased. However, there was no correlation (*p* > 0.05) with caries self-report and poverty in middle schoolchildren (Table 2). When we analyzed caries jointly for elementary + middle children, we observed a positive correlation with both poverty and extreme poverty: when they increased, caries self-report also increased.

A positive correlation was observed between caries prevalence in elementary and middle schoolchildren (r = 0.4199, *p* = 0.0167): when prevalence of caries self-report increased in elementary school students, caries self-report increased in middle school students.

## 4. Discussion

The present study aimed to determine whether there is an association at the ecological level between dental caries self-report in Mexican elementary and middle schoolchildren and various socioeconomic indicators. The hypothesis that the prevalence of caries in schoolchildren in each state is associated with diverse socioeconomic indicators was partially fulfilled, since a correlation of caries with poverty and extreme poverty was observed. This finding partially fits with the “social gradient in health” and refers to the inverse associations observed between socioeconomic status and mortality/morbidity; that is, the risk of poor health tends to increase with decreasing socioeconomic status [49]. Over the past few decades, there has been an improved understanding of the social foundations of dental health [50]. Health inequalities are becoming a more explicit priority for governments and health systems; however, a social gradient with an incremental reduction in dental health remains to be fully characterized. This is likely due to the nature and mechanics of the gradients are highly variable, often depending on the measures used [51]. Our results support this variation.

Since oral diseases are among the most prevalent diseases worldwide and may significantly reduce quality of life [4,52], it is important to adequately characterize risk factors associated with greater burden of disease [53]. How socioeconomic inequalities in health play out is not straightforward, as there is no single “best” measure of dental health or “best” socioeconomic indicator; the approach may depend on a number of factors, such as the target population or the condition of interest. In addition, each variable may be more or less relevant in different life stages. By using multiple indicators of socioeconomic position, different gradients of association may be found: through examining them in greater detail, a variety of possible mechanisms leading to inequality may be identified [51,54,55]. This was the case in our study, as we did not find a relationship between caries self-report and indicators of deprivation nor the Gini index. This could be due to the fact that ecological socioeconomic indicators measure different aspects, as other authors have proposed for indicators such as education, occupation, current income, housing conditions, and current place where one lives, among others [56,57,58,59,60,61]. Conceptually, individual and area socioeconomic characteristics do not necessarily belong to the same constructs, and thus, they may be affecting health through diverse mechanisms [59]. Our results are aligned with prior research reports from Mexico [6,7,38] and other countries [23,25,27,28,29,30,31] where socioeconomic inequalities have been associated to oral health indicators. Studies on dental caries inequalities in low- and middle-income countries are relatively scarce [25], and there are even fewer studies about the spatial distribution pertaining specifically to caries [29]. The one consistent finding across this body of knowledge (individual and ecologic) is that people at the poorer end of the socioeconomic spectrum suffer from larger dental caries impacts.

A clear relationship between the socioeconomic position and various health responses still is elusive: various mechanisms have been proposed to explain such association, including biological issues such as inflammatory biomarkers, DNA or RNA-based markers, indicators of physiological functioning, and modulation of immune system response via latent infections [62,63,64]. Although each of these associations has a different set of confounding factors to be taken into account, a dose–response relationship is consistently observed [62,63,64]. It has been proposed that adverse social exposures trigger neuroendocrine and immune responses, leading to disease and/or increased susceptibility to disease [50]. Research in the social epidemiology of oral health has quantified the contribution of various sociopolitical, social, and health system factors to a range of dental conditions [50]. Such factors include the influence of exposures within the community where one lives [29,65,66,67], e.g., prevalent low levels of education. Since a combination of community and individual effects is possible [65], some researchers argue that health may be affected by unhealthy lifestyles and behaviors as well as access to poor quality services [68].

The present study has limitations that must be taken into account when interpreting its results. First, being an ecological study, its design presents the potential problem called “ecological fallacy”, which is the risk of making an inference at the individual level (that is, about inter-individual variability) from data at the group level [69]. Second, there is the possibility of endogeneity of data, which is given to the narrow age range included and circumscribing the target population solely to the national public educational system. Finally, it is not possible to infer causality from factors included in the analysis; present results should be interpreted as statistical associations.

## 5. Conclusions

In conclusion, the results suggest the presence of socioeconomic inequalities in dental caries self-report in Mexican schoolchildren. At the ecological level in Mexican schoolchildren, not all socioeconomic indicators correlated significantly with caries self-report, but well-established evaluations of poverty and extreme poverty in fact did hold a strong relationship. Public programs to improve the oral health and caries prevention in schoolchildren must be objectively evaluated on a continuous basis to fully ascertain their impacts; subsequent interventions (e.g., fluoride varnish, pit and fissure sealants) ought to be considered should their need be apparent to further reduce socioeconomic inequalities associated with dental caries experience.

## Figures and Tables

**Table 1:** State prevalence of caries self-reported in elementary schoolchildren in Mexico.

State	Sample	Caries				%Poverty 2008	%Poverty 2010	%ExtPov 2008	%ExtPov 2010	GINI 2010
		Yes	No	Not Know	SMI					
Aguascalientes	1183	45.1	49.7	5.1	−0.91086	37.6	38.1	4.2	3.8	0.5074
Baja California	1347	47.9	44.1	8.0	−1.14015	26.0	31.5	3.3	3.4	0.5058
Baja California Sur	1035	46.1	42.1	11.7	−0.68129	21.4	31.0	2.7	4.6	0.4853
Campeche	879	44.5	48.1	7.4	0.43357	45.9	50.5	11.9	13.8	0.5139
Coahuila	1193	58.0	37.6	4.4	−1.14000	32.7	27.8	3.1	2.9	0.4761
Colima	939	43.2	45.9	10.9	−0.77858	27.4	34.7	1.7	2.5	0.4200
Chiapas	689	25.9	60.2	13.9	2.31767	77.0	78.5	38.7	38.3	0.5409
Chihuahua	1172	45.6	44.8	9.6	−0.51977	32.1	38.8	6.7	6.6	0.4719
Ciudad de México	999	46.3	49.3	4.4	−1.48228	27.6	28.5	2.1	2.2	0.5172
Durango	969	48.0	49.1	2.9	0.05248	48.4	51.6	11.5	10.5	0.4699
Guanajuato	1153	37.0	46.7	16.3	0.06075	44.1	48.5	7.9	8.4	0.4331
Guerrero	967	47.0	47.8	5.3	2.53246	68.4	67.6	32.4	31.8	0.5157
Hidalgo	1110	54.3	41.4	4.3	0.66143	55.2	54.7	15.3	13.5	0.4652
Jalisco	1464	48.6	45.4	6.0	−0.82456	36.7	37.0	4.4	5.3	0.4611
México	1257	59.7	36.5	3.8	−0.55372	43.6	42.9	6.9	8.6	0.4679
Michoacan	1071	63.0	32.9	4.1	0.52584	55.5	54.7	15.4	13.5	0.4889
Morelos	978	53.2	42.9	3.9	−0.27213	48.8	43.2	8.7	6.9	0.4199
Nayarit	1279	54.7	41.6	3.8	0.12183	41.7	41.4	6.2	8.3	0.4876
Nuevo Leon	1046	48.9	38.6	12.6	−1.38323	21.4	21.0	2.6	1.8	0.4975
Oaxaca	1081	44.4	32.3	23.3	2.14624	61.8	67.0	28.3	29.2	0.5087
Puebla	678	56.9	39.2	3.9	0.71224	64.6	61.5	19.0	17.0	0.4813
Queretaro	1062	36.5	58.8	4.8	−0.26398	35.2	41.4	5.5	7.4	0.4874
Quintana Roo	1208	44.1	47.0	8.9	−0.41774	33.7	34.6	7.7	6.4	0.4769
San Luis Potosi	901	52.0	43.4	4.6	0.56416	50.9	52.4	15.4	15.3	0.5071
Sinaloa	992	39.4	51.5	9.2	−0.26018	32.4	36.7	4.6	5.5	0.4660
Sonora	939	32.4	55.7	11.9	−0.70347	27.1	33.1	4.4	5.1	0.4787
Tabasco	757	55.2	40.1	4.8	0.47240	53.8	57.1	15.8	13.6	0.4779
Tamaulipas	1120	40.0	54.3	5.7	−0.72144	33.8	39.0	4.8	5.5	0.4488
Tlaxcala	1142	58.4	36.6	4.9	−0.14984	59.6	60.3	9.5	9.9	0.4250
Veracruz	959	47.4	51.6	1.1	1.07546	51.2	57.6	16.8	18.8	0.5329
Yucatan	1210	41.2	49.8	9.0	0.42295	47.0	48.3	8.9	11.7	0.4623
Zacatecas	986	54.2	37.5	8.3	0.10373	50.1	60.2	9.5	10.8	0.5212

ExtPov = extreme poverty. SMI = State Deprivation Index. Caries in elementary school children vs. poverty 2008: r = 0.5336; *p* = 0.0017. Caries in elementary school children vs. poverty 2010: r = 0.4858; *p* = 0.0048. Caries in elementary school children vs. extreme poverty 2008: r = 0.4239; *p* = 0.0156. Caries in elementary school children vs. extreme poverty 2010: r = 0.3952; *p* = 0.0252.

**Table 2:** State prevalence of caries self-reported in middle schoolchildren in Mexico.

State	Sample	Caries Self- Reported			SMI	%Poverty 2008	%Poverty 2010	%ExtPov 2008	%ExtPov 2010	GINI 2010
		Yes	No	Not Know						
Aguascalientes	575	44.1	49.6	6.4	−0.91086	37.6	38.1	4.2	3.8	0.5074
Baja California	883	36.5	50.4	13.1	−1.14015	26.0	31.5	3.3	3.4	0.5058
Baja California Sur	759	40.6	48.3	11.1	−0.68129	21.4	31.0	2.7	4.6	0.4853
Campeche	569	27.2	58.2	14.5	0.43357	45.9	50.5	11.9	13.8	0.5139
Coahuila	685	35.4	58.1	6.6	−1.14000	32.7	27.8	3.1	2.9	0.4761
Colima	660	31.4	58.9	9.7	−0.77858	27.4	34.7	1.7	2.5	0.420
Chiapas	712	37.3	56.3	6.4	2.31767	77.0	78.5	38.7	38.3	0.5409
Chihuahua	776	36.2	51.7	12.1	−0.51977	32.1	38.8	6.7	6.6	0.4719
Ciudad de México	245	35.8	56.9	7.3	−1.48228	27.6	28.5	2.1	2.2	0.5172
Durango	654	35.8	56.8	7.4	0.05248	48.4	51.6	11.5	10.5	0.4699
Guanajuato	865	24.9	63.1	12.0	0.06075	44.1	48.5	7.9	8.4	0.4331
Guerrero	889	35.8	54.8	9.4	2.53246	68.4	67.6	32.4	31.8	0.5157
Hidalgo	717	49.5	43.9	6.5	0.66143	55.2	54.7	15.3	13.5	0.4652
Jalisco	617	33.3	56.9	9.7	−0.82456	36.7	37.0	4.4	5.3	0.4611
México	532	42.4	52.2	5.4	−0.55372	43.6	42.9	6.9	8.6	0.4679
Michoacan	495	38.8	54.0	7.2	0.52584	55.5	54.7	15.4	13.5	0.4889
Morelos	646	42.6	51.6	5.8	−0.27213	48.8	43.2	8.7	6.9	0.4199
Nayarit	544	30.1	63.0	6.9	0.12183	41.7	41.4	6.2	8.3	0.4876
Nuevo Leon	802	28.1	66.8	5.1	−1.38323	21.4	21.0	2.6	1.8	0.4975
Oaxaca	1015	34.7	56.5	8.9	2.14624	61.8	67.0	28.3	29.2	0.5087
Puebla	848	38.2	52.6	9.2	0.71224	64.6	61.5	19.0	17.0	0.4813
Queretaro	546	38.7	52.6	8.7	−0.26398	35.2	41.4	5.5	7.4	0.4874
Quintana Roo	1067	29.2	57.1	13.7	−0.41774	33.7	34.6	7.7	6.4	0.4769
San Luis Potosi	638	33.9	58.3	7.8	0.56416	50.9	52.4	15.4	15.3	0.5071
Sinaloa	649	35.0	58.4	6.6	−0.26018	32.4	36.7	4.6	5.5	0.466
Sonora	622	36.4	55.8	7.8	−0.70347	27.1	33.1	4.4	5.1	0.4787
Tabasco	278	42.2	52.0	5.8	0.47240	53.8	57.1	15.8	13.6	0.4779
Tamaulipas	860	25.4	63.3	11.3	−0.72144	33.8	39.0	4.8	5.5	0.4488
Tlaxcala	573	51.4	46.0	2.7	−0.14984	59.6	60.3	9.5	9.9	0.425
Veracruz	433	27.3	69.1	3.6	1.07546	51.2	57.6	16.8	18.8	0.5329
Yucatan	686	31.5	56.6	11.9	0.42295	47.0	48.3	8.9	11.7	0.4623
Zacatecas	752	41.8	53.0	5.2	0.10373	50.1	60.2	9.5	10.8	0.5212

ExtPov = extreme poverty. SMI = State Deprivation Index. Caries in middle school children vs. poverty 2008: r = 0.1958; *p* = 0.2827. Caries in middle school children vs. poverty 2010: r = 0.1315; *p* = 0.4732. Caries in middle school children vs. extreme poverty 2008: r = 0.0077; *p* = 0.9666. Caries in middle school children vs. extreme poverty 2010: r = −0.0264; *p* = 0.8859.

**Table 3:** State prevalence of caries self-report in elementary and middle schoolchildren in Mexico.

State	Sample	Caries Self- Reported			SMI	%Poverty 2008	%Poverty 2010	%ExtPov 2008	%ExtPov 2010	GINI 2010
		Yes	No	Not Know						
Aguascalientes	1758	44.8	49.7	5.5	−0.91086	37.6	38.1	4.2	3.8	0.5074
Baja California	2230	45.5	45.4	9.1	−1.14015	26.0	31.5	3.3	3.4	0.5058
Baja California Sur	1794	45.0	43.4	11.6	−0.68129	21.4	31.0	2.7	4.6	0.4853
Campeche	1448	39.8	50.9	9.4	0.43357	45.9	50.5	11.9	13.8	0.5139
Coahuila	1878	53.6	41.6	4.8	−1.14000	32.7	27.8	3.1	2.9	0.4761
Colima	1599	40.1	49.4	10.6	−0.77858	27.4	34.7	1.7	2.5	0.420
Chiapas	1401	29.4	59.0	11.6	2.31767	77.0	78.5	38.7	38.3	0.5409
Chihuahua	1948	43.2	46.6	10.3	−0.51977	32.1	38.8	6.7	6.6	0.4719
Ciudad de México	1244	44.8	50.4	4.8	−1.48228	27.6	28.5	2.1	2.2	0.5172
Durango	1623	44.5	51.3	4.2	0.05248	48.4	51.6	11.5	10.5	0.4699
Guanajuato	2018	33.8	51.1	15.2	0.06075	44.1	48.5	7.9	8.4	0.4331
Guerrero	1856	43.7	49.8	6.5	2.53246	68.4	67.6	32.4	31.8	0.5157
Hidalgo	1827	53.3	41.9	4.7	0.66143	55.2	54.7	15.3	13.5	0.4652
Jalisco	2081	44.4	48.6	7.0	−0.82456	36.7	37.0	4.4	5.3	0.4611
México	1789	56.3	39.5	4.1	−0.55372	43.6	42.9	6.9	8.6	0.4679
Michoacan	1566	56.4	38.7	4.9	0.52584	55.5	54.7	15.4	13.5	0.4889
Morelos	1624	50.1	45.4	4.5	−0.27213	48.8	43.2	8.7	6.9	0.4199
Nayarit	1823	47.5	47.9	4.7	0.12183	41.7	41.4	6.2	8.3	0.4876
Nuevo Leon	1848	43.0	46.6	10.5	−1.38323	21.4	21.0	2.6	1.8	0.4975
Oaxaca	2096	41.7	38.9	19.4	2.14624	61.8	67.0	28.3	29.2	0.5087
Puebla	1526	50.3	44.0	5.8	0.71224	64.6	61.5	19.0	17.0	0.4813
Queretaro	1608	37.0	57.3	5.7	−0.26398	35.2	41.4	5.5	7.4	0.4874
Quintana Roo	2275	39.4	50.2	10.4	−0.41774	33.7	34.6	7.7	6.4	0.4769
San Luis Potosi	1539	47.9	46.7	5.3	0.56416	50.9	52.4	15.4	15.3	0.5071
Sinaloa	1641	38.1	53.5	8.4	−0.26018	32.4	36.7	4.6	5.5	0.466
Sonora	1561	33.5	55.7	10.8	−0.70347	27.1	33.1	4.4	5.1	0.4787
Tabasco	1035	52.2	42.8	5.0	0.47240	53.8	57.1	15.8	13.6	0.4779
Tamaulipas	1980	35.2	57.3	7.5	−0.72144	33.8	39.0	4.8	5.5	0.4488
Tlaxcala	1715	57.1	38.4	4.5	−0.14984	59.6	60.3	9.5	9.9	0.425
Veracruz	1392	41.2	56.9	1.8	1.07546	51.2	57.6	16.8	18.8	0.5329
Yucatan	1896	38.8	51.5	9.7	0.42295	47.0	48.3	8.9	11.7	0.4623
Zacatecas	1738	52.3	39.9	7.8	0.10373	50.1	60.2	9.5	10.8	0.5212

ExtPov = extreme poverty. SMI = State Deprivation Index. Caries in elementary and middle school children vs. poverty 2008: r = 0.4973; *p* = 0.0038. Caries in elementary and middle school children vs. poverty 2010: r = 0.4561; *p* = 0.0087. Caries in elementary and middle school children vs. extreme poverty 2008: r = 0.3605; *p* = 0.0427. Caries in elementary and middle school children vs. extreme poverty 2010: r = 0.3364; *p* = 0.0597.

## Data Availability

The datasets generated and analyzed are available from the corresponding author on a reasonable request.
